# Bronchodilator Responsiveness and Reported Respiratory Symptoms in an Adult Population

**DOI:** 10.1371/journal.pone.0058932

**Published:** 2013-03-15

**Authors:** Wan C. Tan, Jean Bourbeau, Paul Hernandez, Kenneth R. Chapman, Robert Cowie, J. Mark FitzGerald, Shawn Aaron, Darcy D. Marciniuk, Francois Maltais, A. Sonia Buist, Denis E. O’Donnell, Don D. Sin

**Affiliations:** 1 UBC James Hogg Research Laboratories, Providence Heart+Lung Institute, St. Paul’s Hospital, University of British Columbia, Vancouver, British Columbia, Canada; 2 Respiratory Epidemiology and Clinical Research Unit, Montreal Chest Institute, McGill University, Montréal, Quebec, Canada; 3 Respiratory Division, Department of Medicine, QEII Health Sciences Centre, Dalhousie University, Halifax, Nova Scotia, Canada; 4 Asthma & Airway Centre, University Health Network and University of Toronto, Toronto, Ontario, Canada; 5 Departments of Medicine and Community Health Sciences, University of Calgary, Calgary, Alberta, Canada; 6 University of British Columbia, Institute for Heart and Lung Health, Vancouver, British Columbia, Canada; 7 Ottawa Hospital Research Institute, University of Ottawa, Ottawa, Ontario, Canada; 8 Division of Respirology, Critical Care and Sleep Medicine, and Airway research Group, University of Saskatchewan, Saskatoon, Saskatchewan, Canada; 9 Centre de Pneumologie, Institute Universitaire de Cardiologie et de Paneumologie de Quebec, Quebec City, Quebec, Canada; 10 Oregon Health and Science University, Portland, Oregon, United States of America; 11 Division of Respiratory & Critical Care Medicine, Queen’s University, Kingston, Ontario, Canada; University of California San Francisco, United States of America

## Abstract

**Background:**

The relationship between patient-reported symptoms and objective measures of lung function is poorly understood.

**Aim:**

To determine the association between responsiveness to bronchodilator and respiratory symptoms in random population samples.

**Methods:**

4669 people aged 40 years and older from 8 sites in Canada completed interviewer-administered respiratory questionnaires and performed spirometry before and after administration of 200 ug of inhaled salbutamol. The effect of anthropometric variables, smoking exposure and doctor-diagnosed asthma (DDA) on bronchodilator responsiveness in forced expiratory volume in 1 second (FEV_1_) and in forced vital capacity (FVC) were evaluated. Multiple logistic regression was used to test for association between quintiles of increasing changes in FEV_1_ and in FVC after bronchodilator and several respiratory symptoms.

**Results:**

Determinants of bronchodilator change in FEV_1_ and FVC included age, DDA, smoking, respiratory drug use and female gender [p<0.005 to p<0.0001 ]. In subjects without doctor-diagnosed asthma or COPD, bronchodilator response in FEV_1_ was associated with wheezing [p for trend<0.0001], while bronchodilator response for FVC was associated with breathlessness. [p for trend <0.0001].

**Conclusions:**

Bronchodilator responsiveness in FEV_1_ or FVC are associated with different respiratory symptoms in the community. Both flow and volume bronchodilator responses are useful parameters which together can be predictive of both wheezing and breathlessness in the general population.

## Introduction

Bronchodilator responsiveness is often quantified as part of the pulmonary function evaluation of patients with suspected pulmonary disease [1], [2] and has been advocated as a case-finding tool for obstructive lung disease [3] It has been much studied and discussed in patients with chronic obstructive lung disease (COPD [2], [4] but its determinants [5], interpretation [1], [6], [7], and clinical relevance [4], [8] remain unclear.

Although bronchodilator responsiveness has been studied extensively in patients with established COPD, there are relatively few studies of the bronchodilator response in normal persons [9], [10], [11] and a paucity of information on the determinants of bronchodilator responsiveness in the general population [5]. Clearly, a better understanding of what occurs in subjects with little or no airflow obstruction could provide a better understanding of the responses seen in disease [2].

To date, most studies have focused on changes in FEV_1_ while changes in FVC are seldom considered when classifying subjects with reversible or irreversible disease [12], [13]. Nonetheless, respiratory guidelines [1] have recommended using both FEV_1_ or FVC as physiological endpoints to define bronchodilator responsivenss as it has long been appreciated from lung function testing that some subjects may show greater improvement in FVC than in FEV_1_ after administration of bronchodilator. [14], [15] Recent COPD clinical trials [12], [13] have reported that changes in FVC occur in patients who do not show changes in FEV_1_. There is little information on the bronchodilator response in forced vital capacity in a general population [11] and none on its relationship with respiratory symptoms.

The objectives of this analysis were to assess the effect of demographic and subject characteristics as determinants of flow-based and volume-based bronchodilator responsiveness and to examine whether these responses are independently linked to respiratory symptoms in the population.

## Methods

### Ethics Statement

All participants gave written informed consent and the study was approved by the respective university and institutional ethical review boards :UBC/PHC Research Ethics Board, P05-006 (Vancouver); Biomedical-C Research Ethics Board, BMC-06-002(Montreal); UHN REB, 06-0421-B (Toronto); Capital Health Research Ethics Board, CDHA-RS/2007-255 (Halifax); Conjoint Health Research Ethics Board, ID21258 (Calgary); DMED-1240-09 (Kingston); 2009519-01H (Ottawa); Bio-REB09-162(Saskatoon); CER20459 (Quebec City).

Data used for the present study were collected between August 2005 to May 2009, in a large cross-sectional multisite, nation-wide, population-based study on lung health, which constituted the first phase of the Canadian Cohort of Obstructive Lung Disease, CanCOLD study. The study was initiated in Vancouver as part of the BOLD study [16] and then completed in 7 other Canadian cities. The sampling strategy and baseline study protocol of the CanCOLD study were the same as that used in the international Burden of Obstructive Lung Disease [BOLD] initiative, the full details of which has been published elsewhere. [16] Additional specific details of the CanCOLD study were also described in a previous publication. [17].

#### Study population and design

Briefly, random samples of non-institutionalized adults, aged 40 years and older in 8 urban sites (Vancouver, Montreal, Toronto, Halifax, Calgary, Quebec City, Kingston, and Ottawa) were recruited. Random telephone digit dialing [RDD] was used to identify eligible individuals. [16], [17], [18] The creation of the random samples for all sites was undertaken by BC STATS using official census data from Statistics Canada (Survey and Analysis Section; Victoria, BC, Canada) and the recruitment by random digit dialing conducted by NRG Research group (Vancouver, BC, Canada). Eligible individuals were then invited to attend a clinic visit to complete interviewer-administered respiratory questionnaires and to perform pre and post-bronchodilator spirometry. The mean clinic visit participation rate was 74% (range 63–87%). [17].

### Study Questionnaire and Spirometric Measurements

Trained technicians administered questionnaires to participants and performed the spirometric measurements. [16], [17] The standardized core questionnaire was used to elicit respiratory symptoms (chronic cough, sputum, wheezing without cold, shortness of breath).[items shown in Appendix S1] Information on respiratory diagnoses, smoking history, exposure to potential risk factors, current use of respiratory medications (any medication for breathing including nasal decongestant), co-morbidities, health care utilization and health status was also gathered. Spirometry was performed in the absence of respiratory infection within the previous 4 weeks, with the subject in a seated posture, using the EasyOne spirometer (ndd Medical Technologies, Andover, MA, USA) before and 15 min after administration of two puffs (200 µg) of salbutamol via a metered-dose inhaler with a spacer. Pulmonary function quality assurance with over reading was conducted in which all spirograms were reviewed and graded according to ATS/ERS standards [1] with prompt feedback to the technician at each site. The maintenance of quality assurance for spirometry is detailed in previous publications. [16], [17] Only spirometric data that fulfilled the ATS acceptability and repeatability criteria [19] were used for analyses.

### Bronchodilator Reversibility

Three indices of bronchodilator responsiveness were computed for FEV_1_ and for FVC: absolute change from pre-bronchodilator value = absolute difference between pre- and post- values (ΔFEV_1_ and ΔFVC in ml); percentage change relative to pre-bronchodilator value = the difference between pre- and post- values over the pre- value and expressed as a % (%ΔFEV_1_i and %ΔFVCi) ) [1]; percentage change relative to predicted = the difference between pre- and post- values over the predicted value and expressed as a % (%ΔFEV_1_ p and %ΔFVCp). [20] In this analysis, we did not use pre-defined cut-off thresholds [1] to categorize positive or negative bronchodilator response status. Instead, the whole range of post-bronchodilator change was evaluated, either as a continuous variable or in quintiles.

### Statistical Analysis

All data analyses were performed using statistical software (Statistical Analysis Software, version 9.1; SAS Institute; Cary, NC). All tests were two-tailed in nature; we considered a p value of 0.05 or less to be significant.

Descriptive statistics are shown as percentages for categorical data and means and standard deviation [SD] for continuous variables, unless otherwise stated. To address the determinants or predictors of bronchodilator response [BDR], univariate and multivariate linear regression models were constructed with age, body mass index (BMI), gender, ever smoker, the use of respiratory drugs (any medication for breathing including nasal decongestant within the last 12 months), site of study, and current self-reported asthma diagnosis as independent variables and bronchodilator response in forced expiratory volume in one second in 3 indices (ΔFEV_1_, % ΔFEV_1_i, %ΔFEV_1_p) and the corresponding indices for post-bronchodilator response in forced vital capacity (ΔFVC, % ΔFVCi, %ΔFVCp) as dependent continuous variables. Standardized mean estimates were computed for all independent variables to facilitate comparisons among variables with different units, as the values represent the expected changes in post-bronchodilator response per 1 SD increase in the independent variable.

In the logistic regression analyses to explore association between respiratory symptoms and bronchodilator response, we excluded subjects with a pre-existing doctor diagnosis of ever-asthma, COPD, emphysema or chronic bronchitis from the cohort, in order to avoid confounding by pre-existing clinically diagnosed airway disease and its management. Four main respiratory symptoms were analyzed: breathlessness defined as “troubled by shortness of breath when hurrying on the level of walking up a slight hill”; “wheeziness” as “wheezing or whistling in your chest at any time in the last 12 months”; and chronic cough and chronic phlegm as the presence of “cough or phlegm when not having a cold” and “on most days for three months each year” [relevant questionnaire items shown in appendix S1]. As we could not be assured of the linearity of the relationship between symptoms and bronchodilator response, we used quintiles of post-bronchodilator change in FEV_1_ and FVC in the logistic regression models. The odds ratio (OR, 95%CI) for each respiratory symptoms with increasing quintiles of BDR, was referenced to the first quintile and adjusted for age, body mass index (BMI), gender, smoking status, respiratory drugs use, study site, and proportion of Caucasian in the sample.

## Results

### Demographics, Baseline Lung Function and Respiratory Symptoms

Completed questionnaires and spirometry was available for 4669 individuals. Pre- and post-bronchodilator spirometric values which satisfied the ATS acceptability and repeatability criteria were available in 4405 (94%) individuals for FEV_1_ and in 4256 (91%) for FVC and were used in the analysis in the study. In the whole study population, the mean (sd) age was 57.5(11.3) years for men and 57.2(10.9) years for women; 59% of men and 50% of women were ever-smokers, and 15% men and 13% women were current smokers. The FEV_1%_ predicted, mean (sd) was 92.7% (17.6) in men and 92.1% (17.4) in women, while FVC% predicted was 96.7% (15.5) in men and 96.6% (15.6) in women. Twenty nine percent of the study population reported wheezing in the last 12 months, 26% reported breathlessness on hurrying or walking up a slight hill, 12% reported chronic cough on most days for 3 or more months each year, and 10% reported similarly defined chronic phlegm. More women than men reported exertional breathlessness and chronic cough [31% versus 20%, p<0.0001 and 14% versus 10% respectively, p<0.0003]. There were site to site differences in population demographics, lung function, respiratory symptoms and smoking status but not pack years of tobacco exposure. Table 1 also compares the two subgroups stratified by a prior diagnosis of airway disease: a relatively ‘healthy’ group without a diagnosis and those with a diagnosis of airway disease. Respiratory symptoms, especially breathlessness were quite prevalent even in those without a diagnosis [Table 1].

**Table 1:** Population demographics and risk factors of individual sites and for whole cohort.

Variables	All Sites	Sample with No Missing Data on Doctor Diagnosis of AO§		
		No AO	With AO	P value
**Total number**	4669	3543	906	
**Gender (Male % of sample)**	43%	45%	33%	<0.0001#
**Age**	57.3 (11.1)	57.3 (11.0)	57.0 (11.1)	0.4294*
**BMI**	27.9 (6.8)	27.5 (5.4)	29.1 (7.0)	<0.0001*
**Smoking Habits**				
Current Smoker	13.9% (0.5)	12.8%(0.6)	17.4%(1.3)	0.0003#
Ever Smoker	54.3% (0.7)	53.1%(0.8)	59.1%(1.6)	<0.0012#
Pack years of tobacco exposure	23.7 (22.9)	22.0 (22.4)	28.3 (22.5)	<0.0001*
**History of Asthma**				
Current asthma	11.7%(0.5)	–	55.9%(1.6)	<0.0001#
Ever-asthma	16.7%(0.5)	–	80.3%(1.3)	<0.0001#
**Use of Respiratory Drugs**	32.6%(0.7)	23.8%(0.7)	67.1%(1.6)	<0.0001#
**Spirometry Results**				
^ &^%Δ FEV1i	3.9%(5.6)	3.4% (5.0)	5.9% (8.0)	<0.0001*
^ &^%Δ FVCi	0.4%(6.5)	−0.03% (6.0)	2.2% (8.2)	<0.0001*
^ ^ †FEV1% predicted	92.3% (17.8)	94.6%(16.0)	83.5%(20.4)	<0.0001*
^ ^ †FVC % predicted	96.7% (15.8)	98.0%(15.3)	92.5%(17.1)	<0.0001*
^ ^ †FEV1/FVC %predicted	95.0% (10.4)	96.2%(8.9)	89.3%(13.5)	<0.0001*
**Resp Symptoms**				
Breathless	26.2% (0.6)	21.8%(0.7)	44.5%(1.7)	<0.0001#
Wheezing	28.6% (0.7)	19.9%(0.7)	63.1%(1.6)	<0.0001#
Chronic Cough	12.1% (0.5)	8.6%(0.5)	26.6%(1.5)	<0.0001#
Chronic Phlegm	9.5% (0.4)	6.5%(0.4)	21.9%(1.4)	<0.0001#

§ Doctor Diagnosis of AO = presence of self reported prior diagnosis of either ever-asthma, or asthmatic bronchitis, or allergic bronchitis, or COPD, or emphysema, or chronic bronchitis. Data for Age, BMI, Packyears, and Spirometry results are expressed in mean(SD); All others are expressed as % of group(SE) and are weighted to the local population. BMI = Body-mass index;

† % predicted values = maximum values/predicted values(NHANES)*100;

* One-Way ANOVA, alpha = 0.05;

# Chi-Square Test.

& post bronchodilator responses: % change in FEV1 or FVC after bronchodilator relative to pre-bronchodilator value.

### Determinants of Bronchodilator Responsiveness in FEV_1_ and FVC

Tables 2 and 3 show the unadjusted and adjusted results from the univariate and multivariate linear analyses for the predictors of bronchodilator responsiveness for FEV_1_ and FVC relative to pre-bronchodialtor value, (%ΔFEV_1_i and %ΔFVCi)in the whole cohort. In Tables 2 and 3, the standard estimate or coefficient for each variable is shown. It is the magnitude of change in bronchodilator response per 1 SD increase in the variable for contiunous variable [age and BMI] or change in status for catogorical variables such as current-asthma diagnosis, respiratory drug use, smoking status. In clinical terms this would mean that Bronchodilator responsiveness (BDR) was less in women than in men [negative coefficient] while the presence of current-asthma, ever-smoking respiratory drug use, increasing BMI and increasing age predicted increasing BDR [positive coefficient]. Before adjustment, predictors for both indices include all covariates, with the exception of female gender for bronchodilator change in FEV_1._ After adjustment for other confounding variables, the most ‘influential’ variables in terms of estimated effect size on BDRFEV1 was doctor diagnosis of current-asthma, followed by age, ever-smoking, use of respiratory drugs, and gender. The same variables affect BDRFVC, with increased importance for gender.

**Table 2:** Determinants of bronchodilator responsiveness in forced expiratory volume in one second as % pre-bronchodilator value [%ΔFEV1i] –results from univariate and multivariate analyses of the whole cohort.

Determinants of bronchodilator response	Univariate [unadjusted]		Multivariate [adjusted]	
	Standardized Estimate	P	Standardized Estimate	P
**Female**	−0.0289	0.0554	−0.0417	0.0051
**Age**	0.0826	<0.0001	0.0954	<0.0001
**Asthma Diagnosis**	0.1629	<0.0001	0.1522	<0.0001
**Use of Respiratory Drugs**	0.1026	<0.0001	0.0496	0.0020
**Ever Smoker**	0.0682	<0.0001	0.0595	<0.0001
**BMI**	0.0401	0.0077	0.0167	0.2649

* Standard estimates allow comparison between variables with different units. It is the expected change in bronchodilator response per 1 SD increase in the variable. After multivariate correction for confounding variables the ‘most powerful’ effect on BDRFEV1 is doctor diagnosis of current-asthma, followed by age, ever-smoking, use of respiratory drugs (any medication for breathing including nasal decongestant), and gender.These values are adjusted for all corivariates including site and for the proportion of Caucasian population in each site.

**Table 3:** Determinants of bronchodilator responsiveness in forced vital capacity as % pre-bronchodilator value [%ΔFVCi] –results from univariate and multivariate analyses of whole cohort.

Determinants of bronchodilator response	Univariate [unadjusted]		Multivariate [adjusted]	
	Standardized Estimate	P	Standardized Estimate	P
**Female**	−0.0479	0.0018	−0.0557	0.0002
**Age**	0.1002	<0.0001	0.1111	<0.0001
**Asthma Diagnosis**	0.1232	<0.0001	0.1145	<0.0001
**Use of Respiratory Drugs**	0.0827	<0.0001	0.0475	0.0039
**Ever Smoker**	0.0627	<0.0001	0.0543	0.0004
**BMI**	0.0466	0.0024	0.0266	0.0822

* Standard estimates allow comparison between variables with different units. It is the expected change in bronchodilator response per 1 SD increase in the variable. After multivariate correction for confounding variables the ‘most powerful’ effect on BDRFVC is doctor diagnosis of current-asthma, followed by age, gender, ever-smoking, and use of respiratory drugs(any medication for breathing including nasal decongestant). These values are adjusted for all corivariates including site and for the proportion of Caucasian population in each site.

Additional analyses of “healthy” individuals excluding those with a prior doctor diagnosis of asthma or COPD, showed similar relationship : for BDRFEV1, the most influential determinants were age[p<0.0001], then ever-smoking[0.0008], then gender[0.024]; for BDRFVC, the significant determinants were age [p<0.0001]and gender[0.003]. When the analyses were repeated using the other indices of bronchodilator change (absolute change and change relative to predicted), the predictors remained unchanged (results not shown).

### Association of Bronchodilator Responsiveness and Respiratory Symptoms

Table 4 and Table 5 summarize the results of the multivariate logistic regression analyses performed to determine the association between respiratory symptoms and increasing quintiles of bronchodilator responsiveness in FEV_1_ and FVC in a cohort not confounded by pre-existing diagnosis of an airway disease and its management. Increasing bronchodilator responsiveness in FEV_1_ was associated with risk for “wheeziness” with significant p for trend. Increasing bronchodilator responsiveness in FVC was associated with reported breathlessness from the second quintile upwards. Similar results for the subgroup with self-reported doctor diagnosis of ever-asthma, COPD, chronic bronchitis and emphysema are shown as Tables S1 and S2.

**Table 4:** Logistic regression analysis of cohort without self-reported diagnosis of ever-asthma, COPD/Chronic bronchitis/Emphysema (n = 3508) showing risk [adjusted Odds ratio & 95% confidence intervals] of Symptoms with increasing post-bronchodilator change in forced expiratory volume in 1 sec % pre-bronchodilator value (%ΔFEV1i).

Quintile*	1	2	3	4	5	P for trend
**Breathlessness**	1	0.88 (0.67–1.16)	1.12 (0.86–1.47)	1.04 (0.79–1.37)	1.24 (0.93–1.64)	0.0770
**Wheeziness**	1	0.87 (0.62–1.10)	0.98 (0.74–1.29)	1.27 (0.97–1.66)	1.63 (1.24–2.15)	<0.0001^#^
**Chronic Cough**	1	0.98 (0.68–1.42)	0.84 (0.57–1.23)	0.86 (0.59–1.27)	1.23 (0.85–1.78)	0.5388
**Chronic Phlegm**	1	0.72 (0.48–1.10)	0.63 (0.41–0.97)	0.72 (0.47–1.10)	0.96 (0.64–1.44)	0.7490

* Variables for the first Quintile were used as the reference;

#Slope for trend was statistically different from the horizontal. Odds ratios and 95% CI adjusted for age, BMI, gender, usage of respiratory drugs (any medication for breathing including nasal decongestant), ever-smoking, site, and porpotion of Caucasian.

**Table 5:** Logistic regression analysis of cohort without self-reported diagnosis of ever-asthma, COPD/Chronic bronchitis/Emphysema (n = 3378) showing risk [adjusted Odds ratio & 95% confidence intervals] of Symptoms with increasing post-bronchodilator change in forced vital capacity as % Pre-bronchodilator value (%ΔFVCi).

Quintile*	1	2	3	4	5	P for trend
**Breathlessness**	1	1.53 (1.15–2.05)	1.53 (1.14–2.06)	1.53 (1.14–2.05)	2.01 (1.50–2.70)	<0.0001^#^
**Wheeziness**	1	1.04 (0.79–1.38)	1.01 (0.76–1.34)	1.08 (0.81–1.43)	1.36 (1.02–1.81)	0.0569
**Chronic Cough**	1	1.27 (0.86–1.86)	1.03 (0.69–1.54)	1.02 (0.69–1.53)	1.31 (0.88–1.94)	0.4954
**Chronic Phlegm**	1	1.15 (0.75–1.78)	0.82 (0.51–1.32)	0.92 (0.59–1.46)	1.64 (1.08–2.49)	0.0874

* Variables for the first Quintile were used as the reference;

#Slope for trend was statistically different from the horizontal. Odds ratios and 95% CI adjusted for age, BMI, gender, usage of respiratory drugs (any medication for breathing including nasal decongestant), ever-smoking, site, and proportion of Caucasian.

These differential associations of bronchodilator responsivenss in FEV_1_ and in FVC with wheeziness and breathlessness on exertion respectively, remain consistent for all three indices of bronchodilator reversibility (absolute change, Δ% initial and Δ% predicted) (data not shown).

## Discussion

The present study provides new information on bronchodilator responsiveness in the population, an area in which there is a paucity of data and understanding. In this report, we have defined the ‘relative importance’ of the determinants of bronchodilator response in FEV_1_ and FVC in a general population aged 40 years and older, and explored the association between increasing bronchodilator responsiveness and respiratory symptoms in the community. The key independent predictors of bronchodilator responsiveness, in descending order of importance, were a doctor diagnosis of current-asthma, age, smoking use of respiratory medications and gender. Conversely, increasing bronchodilator responsiveness in FEV_1_ was consistently associated with wheezing in the last 12 months while increasing bronchodilator responsiveness in FVC was linked to breathlessness on exertion even in the absence of a diagnosis of asthma or COPD. These findings provide novel information and insights on bronchodilator response in unselected adults without a previous diagnosis of airway disease in the general population. The distinct associations between bronchodilator responsiveness and respiratory symptoms further highlight the independent and relative significance of each spirometric measure for assessing bronchodilator responsiveness.

We have shown that bronchodilator responsiveness was positively associated with an increased burden of respiratory symptoms, even in the absence of a diagnosis of asthma or COPD. A conceivable explanation could be that any difference in bronchial responsiveness in normality and in disease is quantitative rather than qualitative. Observations from previous studies of normal subjects and of patients could support this speculation. Bronchodilator responsiveness is normally distributed in healthy subjects [9], [11], [21] and in people with airway disease [6], [22]; the upper 95^th^ percentile cut-off in post-bronchodilator response for both groups were also similar. [11]. Nevertheless, people with asthma and COPD more frequently exceeded the 95^th^ percentile threshold of post-bronchodilator change compared with normal subjects. [11] The alternative explanation could be that we did not fully capture the confounding effect of undetected airway disease or treatment for chronic airway disease that may affect bronchodilator responsiveness in the general population.

The differential association of wheezing with responsiveness in FEV_1_ and of breathlessness with responsiveness in FVC differed from the only published study of healthy subjects from two age cohorts (47–48 years and 71–73 years) which had found that both breathlessness and wheezing were related to a post bronchodilator threshold change in FEV_1_>200 ml and >12% relative to pre-bronchodilator FEV_1_. [10] The authors had cautioned against extrapolating the findings into the general population because of the limited age range of their subjects. [10] We believe we have addressed this concern by using data from multi-sites, population-based samples with wide age range; by using all available data on bronchodilator response and not limiting the analyses to those who exceeded a predefined cut-off threshold for abnormality; and by including FVC responsiveness in the analysis.

Responsiveness in FVC (BDRFVC) continues to be under-recognized and under-utilized in practice, despite the ATS/ERS recommendation of the use of both BDRFEV_1_ and BDRFVC measurements for the evaluation of bronchial reversibility, and even though the occurrence of ‘volume responders’(BDRFVC) have been well documented. [14], [15] In this study, the two measures of bronchial responsiveness would seem to suggest separate clinical relevance, even though both measures were influenced similarly by age, sex, preexisting asthma diagnosis and smoking. Yet, BDRFEV_1_ was associated with wheezing while BDRFVC was a marker of exertional breathlessness. This observation in a general population is consistent with the evidence for the complementary and sometime paradoxical roles for bronchial reversibility in flow and in volume demonstrated in clinical drug trials in COPD patients [12] and in individuals with airflow limitation in the general population [11]. COPD patients may show a heterogenous response in FEV_1_ and FVC; those who do not show bronchial responsiveness in FEV_1_ may show changes in lung volume measurements [12], [23], [24]. In the general population, the proportion of individuals who were above the 95^th^ percentile threshold of BDRFEV_1_ decreased with increasing severity of chronic airway limitation while that for BDRFVC showed the reverse trend [11], a phenomenon also observed in patients with COPD. [12] The heterogeneous responses in FEV_1_ and FVC thus suggested different mechanisms.

Some insight into the association of BDRFVC with breathlessness can be found in clinical studies of the relationship between changes in lung volumes and exercise tolerance [24], [25], [26] and changes in dyspnoea [27] in COPD patients. In patients with COPD and moderate to severe hyperinflation characterized by reduced FVC and inspiratory capacity, five times as many patients showed improvement in FVC as changes in FEV_1_ with bronchodilators. [24]. Improvement in FVC correlated with improvement in exercise tolerance and endurance [25], [26]; and improvement in inspiratory capacity was related to improvement in dyspnoea. [27]. ] Hence, a volume change post-bronchodilator could be a marker of dynamic hyperinflation [13], [26] and premature airway closure [28] both of which result in dyspnoea. It is also conceivable that FVC responsiveness could be a marker of early or sub-clinical COPD, though this speculation could only be clarified by data from further longitudinal follow-up.

This is the first national study to assess BDR in population-based samples from a large number of sites. Other strengths include detailed attention to standardization and quality control used across all sites [16], [17]. The inclusion of all three common expressions for change in post-bronchodilator FEV_1_ [absolute change, relative change to pre-value and to predicted values] and the additional analyses of the FVC data provides a much more comprehensive evaluation than in previous studies of bronchodilator responsiveness in healthy subjects [9], [10], [21].

A potential limitation of the study is that the exclusion of subjects with airway disease was based on self-reported doctor-diagnosed asthma or COPD or emphysema or chronic bronchitis rather than on health records or objective spirometric criteria. This diagnosis- based criteria might not have excluded all with airway obstruction in the ‘healthy subgroup’. Additionally, the physiological underpinnings of increased bronchodilator responsiveness in the general population remain unresolved. It is possible that some subjects with increased responsiveness may represent undiagnosed asthma. Mechanistic and longitudinal studies would be needed to validate this hypothesis. Another limitation was that we used 200 ug of salbutamol/albuterol for bronchodilation instead of higher doses as an extra precaution because it was a field study where many of the subjects were assessed at home. Although higher doses may have potentially augmented the bronchial response, it was unlikely to have altered the findings in this study. Finally, we have identified the determinants and clinical associations of acute reversibility in a single setting but not the variability of these determinants and the relevance of the association with symptoms over time or on long term patient outcomes. Further confirmation and clarification of these cross-sectional findings await data from longitudinal studies.

In summary, the results of this population-based study showed that bronchodilator response is a marker of the undetected burden of respiratory symptoms in the community and highlight the independent significance of flow-based and volume-based spirometric measures of airway reversibility testing, which together are associated with wheezing and breathlessness.

## Supporting Information

Table S1 **Logistic regression analysis for subgroup with self-reported diagnosis of ever-asthma, COPD/Chronic bronchitis/Emphysema (n = 897) showing risk [adjusted Odds ratio & 95% confidence intervals] of Symptoms with increasing post-bronchodilator change in forced expiratory volume in 1 sec % pre-bronchodilator value (%ΔFEV1i).**
(DOC)

Table S2 **Logistic regression analysis for subgroup with self-reported diagnosis of ever-asthma, COPD/Chronic bronchitis/Emphysema (n = 878) showing risk [adjusted Odds ratio & 95% confidence intervals] of Symptoms with increasing post-bronchodilator change in forced vital capacity as % pre-bronchodilator value (%ΔFVCi).**
(DOC)

Appendix S1 **Respiratory Questions used in definition of Respiratory symptoms in** **table 4** **and** **5** **[from standaridized core questionnaire used in study].**
(DOC)
