# Supplementary material for: Bronchodilator Responsiveness and Reported Respiratory Symptoms in an Adult Population
Source: PLoS One. 2013 Mar 15;8(3):e58932. doi: 10.1371/journal.pone.0058932 (PMC3598856; doi:10.1371/journal.pone.0058932)
Supplement: Table S1 — Logistic regression analysis for subgroup with self-reported diagnosis of ever-asthma, COPD/Chronic bronchitis/Emphysema (n = 897) showing risk [adjusted Odds ratio & 95% confidence intervals] of Symptoms with increasing post-bronchodilator change in forced expiratory volume in 1 sec % pre-bronchodilator value (%ΔFEV1i). (DOC) [file pone.0058932.s001.doc]

Table S1

Logistic regression analysis for subgroup with self-reported diagnosis of ever-asthma, COPD/Chronic bronchitis/Emphysema (n=897) showing risk [adjusted Odds ratio & 95% confidence intervals] of Symptoms with increasing post-bronchodilator change in forced expiratory volume in 1 sec % pre-bronchodilator value (%∆FEV1i)

| **Quintile*** | **1** | **2** | **3** | **4** | **5** | **P for trend** |
| --- | --- | --- | --- | --- | --- | --- |
| **Breathlessness** | 1 | 0.86  (0.50-1.49) | 0.55  (0.32-0.96) | 0.84  (0.49-1.42) | 0.74  (0.46-1.20) | 0.3444 |
| **Wheeziness** | 1 | 1.25  (0.75-2.07) | 1.06  (0.64-1.76) | 1.26  (0.77-2.05) | 2.31  (1.45-3.67) | 0.0003# |
| **Chronic Cough** | 1 | 1.07  (0.61-1.88) | 1.27  (0.73-2.21) | 1.06  (0.62-1.82) | 1.21  (0.74-1.97) | 0.5101 |
| **Chronic Phlegm** | 1 | 0.90  (0.49-1.64) | 1.06  (0.59-1.92) | 1.05  (0.59-1.86) | 1.22  (0.73-2.03) | 0.2861 |

***** Variables for the first Quintile were used as the reference; # Slope for trend was statistically different from the horizontal. Odds ratios and 95% CI adjusted for age, BMI, gender, usage of respiratory drugs, ever-smoking, site, and porpotion of Caucasian.
