# Supplementary material for: Bronchodilator Responsiveness and Reported Respiratory Symptoms in an Adult Population
Source: PLoS One. 2013 Mar 15;8(3):e58932. doi: 10.1371/journal.pone.0058932 (PMC3598856; doi:10.1371/journal.pone.0058932)
Supplement: Table S2 — Logistic regression analysis for subgroup with self-reported diagnosis of ever-asthma, COPD/Chronic bronchitis/Emphysema (n = 878) showing risk [adjusted Odds ratio & 95% confidence intervals] of Symptoms with increasing post-bronchodilator change in forced vital capacity as % pre-bronchodilator value (%ΔFVCi). (DOC) [file pone.0058932.s002.doc]

Table S2.

Logistic regression analysis for subgroup with self-reported diagnosis of ever-asthma, COPD/Chronic bronchitis/ Emphysema (n=878) showing risk [adjusted Odds ratio & 95% confidence intervals] of Symptoms with increasing post-bronchodilator change in forced vital capacity as % pre-bronchodilator value (%∆FVCi)

| **Quintile*** | **1** | **2** | **3** | **4** | **5** | **P for trend** |
| --- | --- | --- | --- | --- | --- | --- |
| **Breathlessness** | 1 | 0.98  (0.56-1.70) | 0.77  (0.45-1.32) | 1.22  (0.72-2.08) | 1.22  (0.75-2.00) | 0.2076 |
| **Wheeziness** | 1 | 1.55  (0.93-2.59) | 1.35  (0.83-2.20) | 1.56  (0.95-2.56) | 2.01  (1.27-3.18) | 0.0065# |
| **Chronic Cough** | 1 | 1.36  (0.74-2.49) | 1.17  (0.65-2.12) | 1.88  (1.07-3.32) | 1.84  (1.08-3.12) | 0.0096# |
| **Chronic Phlegm** | 1 | 1.63  (0.85-3.12) | 1.55  (0.83-2.89) | 1.90  (1.03-3.51) | 1.69  (0.95-2.98) | 0.1041 |

***** Variables for the first Quintile were used as the reference; # Slope for trend was statistically different from the horizontal. Odds ratios and 95% CI adjusted for age, BMI, gender, usage of respiratory drugs, ever-smoking, site, and proportion of Caucasian.
