# Supplementary material for: Bronchodilator Responsiveness and Reported Respiratory Symptoms in an Adult Population
Source: PLoS One. 2013 Mar 15;8(3):e58932. doi: 10.1371/journal.pone.0058932 (PMC3598856; doi:10.1371/journal.pone.0058932)
Supplement: Appendix S1 — Respiratory Questions used in definition of Respiratory symptoms in table 4 and 5 [from standaridized core questionnaire used in study]. (DOC) [file pone.0058932.s003.doc]

**Appendix S1.** Respiratory Questions used in definition of Respiratory symptoms in table 4 & 5 [ from standaridized core questionnaire used in study]

## Respiratory Symptoms and Disorders

These questions pertain mainly to your chest. Please answer yes or no if possible. If you are in doubt about whether your answer is yes or no, please answer no.

### Cough

7. Do you usually cough when you don’t have a cold? Yes ❑ 1 15

*[If* ***yes****, continue with Question 7A; If* ***no****, skip to Question 8]* No ❑ 2

7A. Are there months in which you cough on most days? Yes ❑ 1 16

No ❑ 2

*[If* ***yes****, ask both Questions 7B & 7C; If* ***no****, skip to Question 8]*

7B. Do you cough on most days for as much as three Yes ❑ 1 17

months each year? No ❑ 2

7C. For how many years have you had this cough? Less than 2 years ❑ 1 18

2-5 years ❑ 2

More than 5 years ❑

### Phlegm

8. Do you usually bring up phlegm from your chest, or do you usually Yes ❑ 1 19

have phlegm in your chest that is difficult to bring up when you No ❑ 2

don’t have a cold?

*[If* ***yes****, continue with Question 8A; If* ***no****, skip to Question 9]*

8A. Are there months in which you have this phlegm on most Yes ❑ 1 20

days? No ❑ 2

*[If* ***yes****, ask both Questions 8B & 8C; If* ***no****, skip to Question 9]*

8B. Do you bring up this phlegm on most days for as much Yes ❑ 1 21

as three months each year? No ❑ 2

8C. For how many years have you had this phlegm? Less than 2 years ❑ 1 22

2-5 years ❑ 2

More than 5 years ❑ 3

### Wheezing/Whistling

9. Have you had wheezing or whistling in your chest at any Yes ❑ 1 23

time in the last 12 months? No ❑ 2

*[If* ***yes****, ask both Questions 9A & 9B; If* ***no****, skip to Question 10]*

9A. In the last 12 months, have you had this wheezing Yes ❑ 1 24

or whistling only when you have a cold? No ❑ 2

9B. In the last 12 months, have you ever had an attack of wheezing Yes ❑ 1 25 or whistling that has made you feel short of breath? No ❑ 2

### Breathlessness

10. Are you unable to walk due to a condition other than shortness Yes ❑ 1 26

of breath? No ❑ 2

[*If* ***yes*** *to Question 10, please describe this condition on the line below and then skip to*

*Question 12. If* ***no****, go directly to Question 11.]*

Nature of condition(s):

11. Are you troubled by shortness of breath when hurrying on the Yes ❑ 1 27

level or walking up a slight hill? No ❑ 2

*[If yes, ask Question 11A through 11D; If* ***no****, skip to Question 12]*

11A. Do you have to walk slower than people of your age on Yes ❑ 1 28

level ground because of shortness of breath? No ❑ 2

Does not apply ❑ 3

11B. Do you ever have to stop for breath when walking at Yes ❑ 1 29

your own pace on level ground? No ❑ 2

Does not apply ❑ 3

11C. Do you ever have to stop for breath after walking Yes ❑ 1 30

about 100 yards (or after a few minutes) on level No ❑ 2

ground? Does not apply ❑ 3

11D. Are you too short of breath to leave the house or Yes ❑ 1 31

short of breath on dressing or undressing? No ❑ 2

Does not apply ❑ 3
